# Generation of a Novel SORT1×HER2 Bispecific Antibody–Drug Conjugate Targeting HER2-Low-Expression Tumor

**DOI:** 10.3390/ijms242216056

**Published:** 2023-11-07

**Authors:** Weiliang Zhuang, Wei Zhang, Lei Wang, Liping Xie, Jun Feng, Baohong Zhang, Youjia Hu

**Affiliations:** 1 Engineering Research Center of Cell & Therapeutic Antibody, Ministry of Education, School of Pharmacy, Shanghai Jiao Tong University, 800 Dongchuan Road, Shanghai 200240, China; zhuangweiliang@sjtu.edu.cn (W.Z.); lwangph@sjtu.edu.cn (L.W.); 2 China State Institute of Pharmaceutical Industry, 285 Gebaini Road, Shanghai 201203, China; bingdong09@163.com (W.Z.); xieliping@sinopharm.com (L.X.); fengjdmr@163.com (J.F.)

**Keywords:** SORT1, HER2 targeted, bispecific antibody–drug conjugate, internalization, tumor inhibition

## Abstract

Human epidermal growth factor receptor 2 (HER2) is considered an ideal antibody–drug conjugate (ADC) target because the gene is overexpressed in many tumors compared to normal tissues. Multiple anti-HER2 ADCs conjugated with different toxic payloads bring benefits to patients with high HER2 expression. However, HER2-targeted ADC technology needs further optimization to improve its effect for the treatment of patients with low HER2 expression. We hypothesized that bispecific antibody–drug conjugate (bsADC) targeting HER2 and Sortilin-1 (SORT1) would overcome this limitation. SORT1 is a suitable target for pairing with HER2 to generate a bispecific antibody (BsAb) since the gene is co-expressed with HER2 in tumors and possesses rapid internalization. We developed a BsAb (bsSORT1×HER2) that exhibited strong binding and internalization activity on HER2-low-expression tumor cells and facilitated higher HER2 degradation. The bsSORT1×HER2 was further conjugated with DXd to generate a bsADC (bsSORT1×HER2-DXd) that showed strong cytotoxicity on HER2-low-expression tumor cells and antitumor efficacy in an MDA-MB-231 xenograft mice model. These results demonstrated that employment of a SORT1×HER2-targeted bsADC may be promising to improve the antitumor efficacy of HER2-targeted ADC for the treatment of tumors with low HER2 expression.

## 1. Introduction

Currently, there are over 10 ADCs for cancer therapy on the market worldwide. The targets of approved ADCs include, but are not limited to, HER2, trophoblast cell surface antigen-2 (Trop-2), CD30, CD22, CD79b and Nectin-4 [1,2,3,4,5,6]. Among these targets, HER2 is one of the most popular ADC targets in the clinical setting. An overexpression of HER2 is associated with several cancers, particularly breast cancer. There are currently two anti-HER2 ADCs approved by the FDA for the treatment of HER2-positive breast cancer: ado-trastuzumab emtansine (T-DM1) [7] and fam-trastuzumab deruxtecan-nxki (DS8201a) [4]. T-DM1 has shown higher response rates and longer progression-free survival compared to standard chemotherapy in HER2-positive breast cancer patients [8]. DS8201a has also shown promising results in clinical trials, particularly in patients with advanced or metastatic HER2-positive breast cancer who have received prior treatment with other HER2-targeted therapies [7,9]. Furthermore, DS8201a has not only demonstrated greater potency and effectiveness than T-DM1 in preclinical models [10,11], but has also shown antitumor activity in HER2-low breast cancer. Of course, the response rate of DS8201a in patients with HER2-high-expression tumors is better than in those with HER2-low-expression tumors [12]. In addition, DS8201a can cause interstitial lung disease (ILD)/pneumonitis [13], which can be fatal. Therefore, ADCs targeting HER2 still need to be further improved to enhance their efficacy against HER2-low-expression tumors and their safety as well. 

The efficacy of ADCs can be influenced by multiple factors, including antibody selection, linker chemistry, cytotoxic payload, the expression level of the target and internalization rate [14]. Compared with T-DM1, DS8201a has a different cytotoxic payload, linker and drug-to-antibody ratio (DAR) [10]. Both of the above HER2-targeted ADCs employ trastuzumab to specifically target HER2-expressing cancer cells. In addition to cytotoxic payload and linker, the ADC effect and safety profile can also be improved by changing the antibody moiety, which determines the internalization rate and tumor specificity of the ADC [15,16]. Li JY et al. [17] developed a biparatopic antibody targeting two distinct epitopes on HER2 that could induce faster internalization and lysosomal trafficking. After conjugating with the cytotoxic payload, the biparatopic ADC demonstrated superior antitumor activity over T-DM1 and potent antitumor activity in HER2-low-expression cancer cells. Other strategies include the development of a HER2-targeted bispecific antibody (HER2-targeted BsAb) [18]. HER2 has poor internalization [19]. Thus, if the target selected for coupling with HER2 initiates rapid internalization, this would facilitate internalization and lysosomal delivery of HER2-targeted BsAb, which could in turn provide stronger antitumor activity to HER2-targeted bsADC. Previous reports [18,20] proved the concept by co-targeting HER2 with CD63 or PRLR to facilitate the internalization and lysosomal delivery of BsAb and induce superior antitumor activity of bsADCs. In this study, we will use SORT1, a protein transporter, as the target.

SORT1 is a transmembrane protein that regulates protein trafficking by endocytosis [21,22]. It is involved in the sorting and transport of various ligands and receptors, including neurotensin, nerve growth factor (NGF) and epidermal growth factor receptor (EGFR) [22,23,24,25,26]. SORT1 is overexpressed in several types of tumor cells, such as breast, colorectal and ovarian cancer cells [25,27,28]. Its overexpression has been associated with increased cancer cell proliferation, migration and invasion [28,29]. SORT1 plays a crucial role in vasculogenic mimicry formation, which was inhibited by a SORT1-targeted peptide–drug conjugate (TH1902) [29]. Furthermore, TH1902 exerted potent antitumor activity in MDA-MB-231 and HCC-70 murine xenograft models [30], which suggested that SORT1-targeted ADC would also functionate in SORT1-positive tumors. We also found that HER2 and SORT1 were co-expressed on many cancer cells, particularly breast cancer cells.

In this study, we developed a BsAb (bsSORT1×HER2) that can facilitate faster internalization and more lysosomal HER2 degradation than trastuzumab. In addition, we showed that bsSORT1×HER2 ADC possessed better antitumor activity than the DS8201a biosimilar in MDA-MB-231 xenograft mice models. Our results suggest that HER2-targeted bsADCs with faster internalization by binding SORT1 may be a promising approach to enhance the antitumor activity in HER2-low-expression tumors.

## 2. Results

### 2.1. SORT1 and HER2 Co-Expression on Surface of Human Cancer Cell Lines

**Figure 1:**
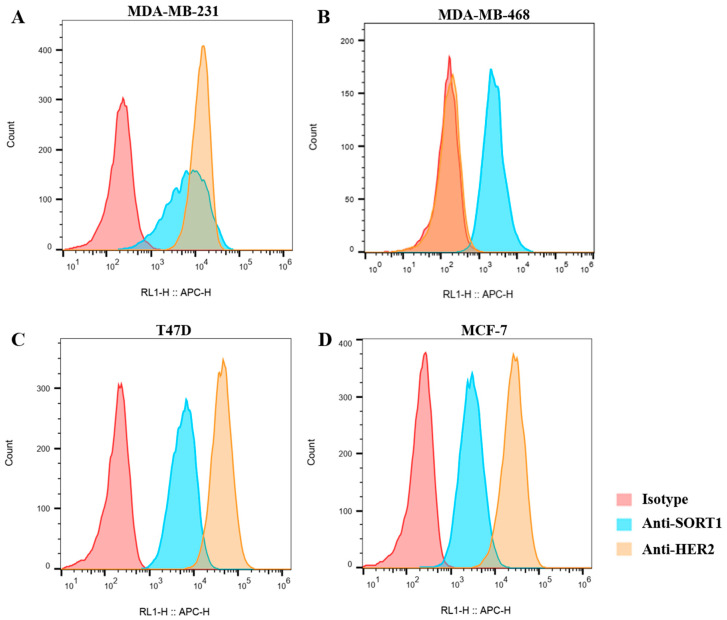
Flow cytometry evaluation of SORT1 and HER2 expression on breast tumor cells. MDA-MB-231 (**A**), MDA-MB-468 (**B**), T47D (**C**) and MCF-7 (**D**).

Cell surface expressions of SORT1 and HER2 in human cell lines were evaluated using flow cytometry. The SORT1/ISO ratios of breast cancer cells MDA-MB-231, MDA-MB-468, T47D and MCF-7 were 33.15, 17.11, 36.31 and 17.11, respectively (Figure 1). This demonstrated that SORT1 may be ubiquitously expressed on the surface of breast cancer cells, in which MDA-MB-231, T47D and MCF-7 were reported as HER2-low-expression cell lines and MDA-MB-468 was reported as a HER2-negative cell line [31,32]. On the contrary, the noncancer breast epithelial cell MCF-10A did not express SORT1 (Table 1). We also detected SORT1 level on cell lines from other tumors, and found that L1NAP (prostate carcinoma), NCI-N87 (gastric carcinoma), SK-OV-3 (ovarian cancer) and A549 (lung carcinoma) expressed relatively lower SORT1 on the surface (Table 1). Unlike SORT1, HER2 expression levels vary widely among different cancer cell lines (Table 1); the HER2/ISO ratios range from 1.1 to 3854.96. According to HER2/ISO ratios, we determined that NCI-N87 and SK-OV-3 were HER2-high-expression cells; MDA-MB-231, T47D and MCF-7 were HER2-low-expression cells; and MDA-MB-468 was a HER2-negative cell. In addition, MDA-MB-231, MDA-MB-468, T47D and MCF-7 were considered to be SORT1-high-expression cells, while L1NAP, NCI-N87, SK-OV-3 and A549 were considered to be SORT1-low-expression cells (Table 1). The HER2/ISO ratios of the HER2-low-/SORT1-high-expression cell lines MDA-MB-231, T47D and MCF-7 were higher than the SORT1/ISO ratios, suggesting that the level of HER2 on the surface of these cell lines was higher than that of SORT1. These results indicated that SORT1 and HER2 were co-expressed on the surface of many cancer cell lines, while the HER2 level on the cell surface was much higher than that of SORT1.

### 2.2. SORT1-Antibody-Induced Faster Internalization than HER2 Antibody

**Figure 2:**
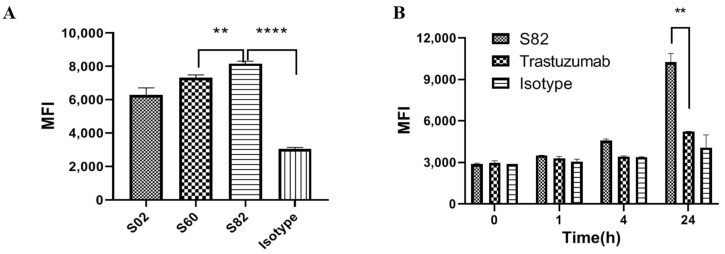
**Internalization function of SORT1 antibodies and trastuzumab:** (**A**) Internalization activity of S02, S60 and S82. T47D cells were treated with pHrodo^TM^ green-labeled antibodies (10 nM) for 24 h. Data are presented as mean ± SD from 3 independent experiments. (**B**) Internalization activity of trastuzumab and S82. Data are presented as mean ± SD from 2 independent experiments. **, *p* < 0.001; ****, *p* < 0.0001.

SORT1 is a transporter that transports proteins from the cell surface to the endosome or lysosome [25]. We assumed that an anti-SORT1 arm of BsAb would transport its partner target into cells and facilitate lysosomal targeting of BsAb. The internalization functions of three currently available anti-SORT1 antibodies were evaluated using flow cytometry. Since the fluorescence of pHrodo^TM^ green would increase dramatically after antibodies were transported into endosome or lysosome, in which the pH was more acidic, the internalization rate of anti-SORT1 antibodies into cells could be identified using fluorescence intensity. As shown in Figure 2A, the MFI of the S82-treated T47D cell was higher than for S02 and S60, which suggested that S82 induced faster lysosome trafficking. Therefore, we selected S82 to be the anti-SORT1 arm for SORT1×HER2 BsAb. In addition, the HER2 antibody trastuzumab was used to provide the anti-HER2 arm of SORT1×HER2 BsAb.

We also compared the internalization activity of S82 and trastuzumab. As shown in Figure 2B, after treating with pHrodo^TM^ green-labeled S82, the MFI of the T47D cell increased rapidly along with the increase in treatment time, while a significant signal was only observed after 24h for trastuzumab-treated cells. This indicated that S82 possessed a faster internalization effect than trastuzumab. Furthermore, the MFI of the S82-treated cell was significantly higher than that of trastuzumab after 24h treatment. This suggested that more antibodies were transported by SORT1 into the lysosome, which should have the benefit of enhancing the cytotoxicity of ADC.

### 2.3. Identification of SORT1×HER2 Bispecific Antibody

**Figure 3:**
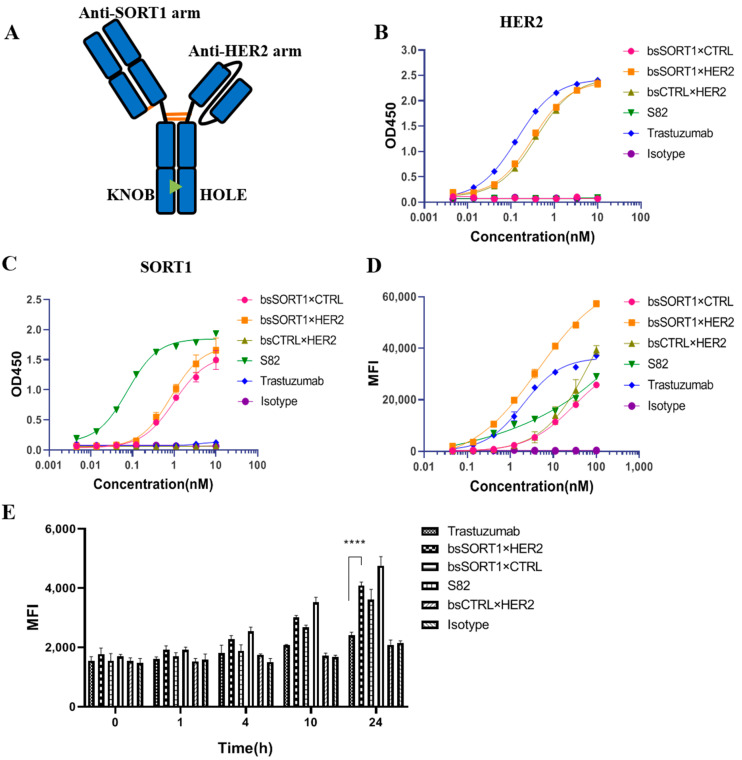
**Characterization of SORT1×HER2 bispecific antibody:** (**A**) The diagram structure of bsSORT1×HER2. (**B**,**C**) The binding activity of bispecific antibodies to HER2 ECD protein (**B**) or SORT1 ECD protein (**C**) were detected via ELISA. EC_50_ values were calculated usin GraphPad Prism software 8. Data are presented as mean ± SD from 2 independent experiments. (**D**)**.** Cell binding of bispecific antibodies was detected via flow cytometry. MDA-MB-231 cells were incubated with diluted antibodies followed by labeling with Alexa Fluor 647-conjugated goat anti-human IgG. MFI values were acquired via flow cytometry and EC_50_ values were calculated using GraphPad Prism software. Data are presented as mean ± SD from 2 independent experiments. **E.** Internalization activity of bispecific antibodies was evaluated using flow cytometry. T47D cells were treated with pHrodo^TM^ green-labeled antibodies for 1, 4, 10 or 24 h followed by detecting MFI of cells. Data are presented as mean ± SD from 3 independent experiments. ****, *p* < 0.0001.

We used “knobs-into-holes” (KIH) technology to generate a BsAb (bsSORT1×HER2), in which the anti-SORT1 arm from S82 is in the form of Fab and the anti-HER2 arm from trastuzumab is in the form of scFv (Figure 3A). As shown in Figure 3B,C, the half-maximal effective concentrations (EC_50_) of bsSORT1×HER2 binding to the SORT1 ECD protein and the HER2 ECD protein were 0.84 nM and 0.32 nM, respectively. The binding activity was equivalent to the corresponding control antibodies bsSORT1×CTRL (EC_50_ = 1.01 nM) or bsCTRL×HER2 (EC_50_ = 0.38 nM). As bsSORT1×HER2 only retained a monovalent SORT1 or HER2 binding domain, its SORT1 binding activity was 11 times weaker than that of S82 (EC_50_ = 0.076 nM) and its HER2 binding activity was 2.3 times weaker than that of trastuzumab (EC_50_ = 0.14 nM). We used flow cytometry to detect the binding activity of bsSORT1×HER2 to the cell co-expressing SORT1 and HER2. As expected (Figure 3D), the binding activity of bsSORT1×HER2 (EC_50_ = 4.34 nM) to MDA-MB-231 was significantly better than that of bsCTRL×HER2 (EC_50_ = 79.3 nM), bsSORT1×CTRL (EC_50_ = 30.4 nM) and S82 (EC_50_ is not available). However, the EC_50_ of bsSORT1×HER2 was higher than that of trastuzumab (EC_50_ = 2 nM), indicating that trastuzumab possessed higher affinity with MDA-MB-231 than bsSORT1×HER2.

In addition, the internalization assay showed that the endocytosis activity induced by bsSORT1×HER2 was significantly better than that of bsCTRL×HER2 and trastuzumab (Figure 3E). Furthermore, bsSORT1×HER2 also possessed faster internalization activity than the corresponding SORT1 antibody bsSORT1×CTRL, indicating that the two target binding arms may have a synergistic effect. 

### 2.4. BsSORT1×HER2 Facilitated More and Faster Degradation of HER2

**Figure 4:**
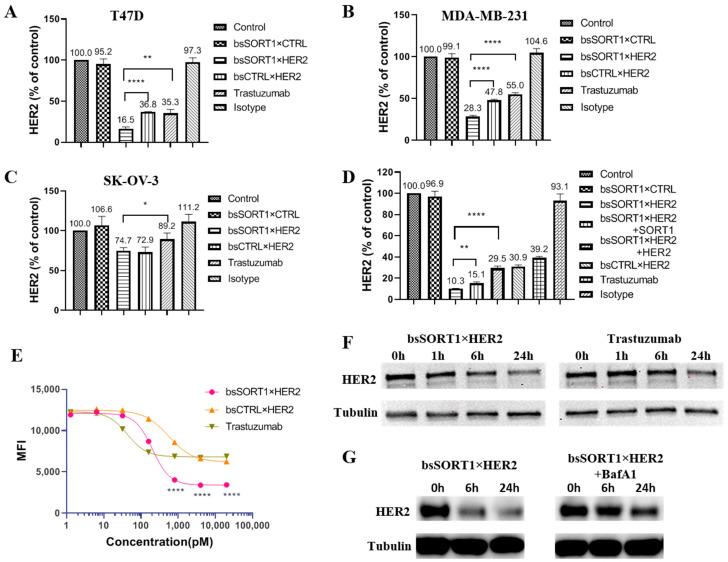
**HER2 degradation by SORT1×HER2 bispecific antibody:** (**A**–**C**) Degradation effects of antibodies on surface HER2 of T47D (**A**), MDA-MB-231 (**B**) and SK-OV-3 (**C**) were detected via flow cytometry. Data were mean ± SD from 3 independent experiments. (**D**) Degradation of surface HER2 was blocked by HER2 ECD protein and SORT1 ECD protein. Data are presented as mean ± SD from 3 independent experiments. (**E**) The degradation was evaluated by treating T47D with serially diluted antibodies (from 9.14 pM to 20 nM). IC_50_ was calculated using GraphPad Prism software 8. Data are presented as mean ± SD from 3 independent experiments. (**F**,**G**) HER2 degradation by antibodies was detected using WB. (**F**) Comparison of the degradation rate of HER2 by bsSORT1×HER2 and trastuzumab. T47D cells were treated with 10 nM bsSORT1×HER2 or trastuzumab for 1, 6 or 24 h. (**G**) The HER2 degradation induced by bsSORT1×HER2 was blocked by lysosome inhibitor. T47D cells were incubated with 10 nM bsSORT1×HER2 in the absence or presence of BafA1 (1 mM). *, *p* < 0.05; **, *p* < 0.001; ****, *p* < 0.0001.

To evaluate HER2 degradation induced by HER2 antibodies, we used FITC-labeled pertuzumab to detect the amount of HER2 remaining on the cell surface after treating with different antibodies. Since the recognizing epitope of pertuzumab is different to that of trastuzumab, trastuzumab does not interfere with the binding of pertuzumab to HER2. The amount of HER2 on the surfaces of T47D, MDA-MB-231 and SK-OV-3 was measured after 24h of incubation with HER2 antibodies (Figure 4A–C). After treating with bsSORT1×HER2, the HER2 level on the surface of T47D was only 16.4% of the control, and lower than that of trastuzumab (35.3%) and bsCTRL×HER2 (36.8%), while bsSORT1×CTRL and the isotype had no effect. Similar results were observed in MDA-MB-231. In the SK-OV-3 cell, with high HER2 and low SORT1 on the surface, bsSORT1×HER2 only induced about 25.3% reduction in surface HER2, which was similar to bsCTRL×HER2. Interestingly, trastuzumab had minimum effect on the amount of surface HER2 in SK-OV-3. As shown in Figure 4D, HER2 or SORT1 extracellular domain (ECD) protein inhibited the reduction in surface HER2 induced by bsSORT1×HER2, proving that bsSORT1×HER2 acted on SORT1 and HER2 simultaneously, resulting in more reduction of surface HER2. Moreover, the decrease in the surface HER2 induced by bsSORT1×HER2 was concentration-dependent (Figure 4E). The half-maximal inhibitory concentration (IC_50_) of bsSORT1×HER2 (IC_50_ = 203.1 pM) was higher than that of trastuzumab (IC_50_ = 42.91 pM), but it had stronger degradation activity than trastuzumab at high concentration.

The degradation of total HER2 was also detected using Western blot. Compared to trastuzumab, bsSORT1×HER2 induced faster and more degradation of HER2 (Figure 4F), which was consistent with its internalization properties. This proved that the co-targeting of SORT1 facilitated the degradation of HER2. Moreover, the effect of bsSORT1×HER2 on HER2 levels was inhibited by BafA1 (Figure 4G), indicating that HER2 degradation induced by bsSORT1×HER2 is a lysosomal process.

### 2.5. BsSORT1×HER2 ADC Showed More Potency in Inhibiting Growth of HER2-Low Cancer Cells In Vitro

**Figure 5:**
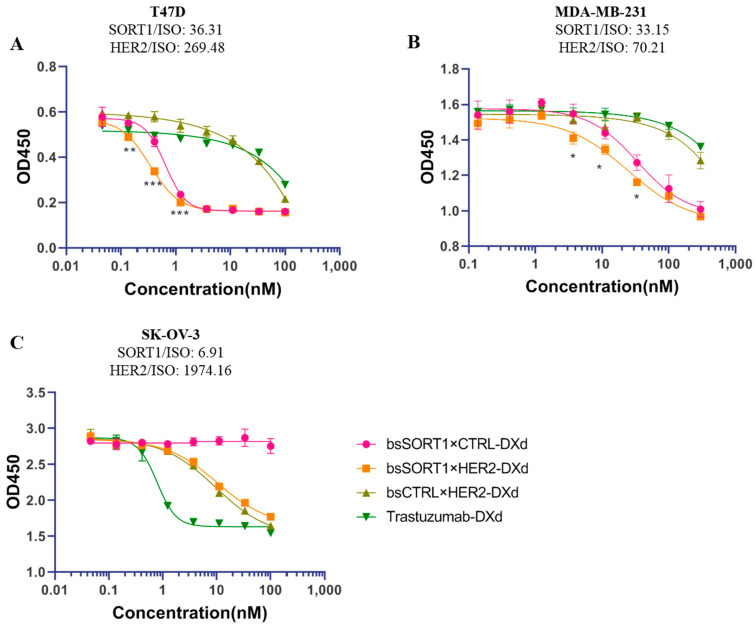
**The cytotoxicity activity of ADCs on T47D (A), MDA-MB-231 (B) and SK-OV-3 (C).** Cells were treated with serially diluted ADCs for 72 hours and cell viability was detected using CCK8. Data are presented as mean ± SD from 3 independent experiments and IC_50_ was calculated using GraphPad Prism software. P values were only calculated between bsSORT1×HER2-DXd and bsSORT1×CTRL-DXd using *t*-test analysis and their significances were shown in the figure. *, *p* < 0.05; **, *p* < 0.001; ***, *p* < 0.0005.

To investigate whether bsADCs have superior inhibitory activity against cell growth, we conjugated bsSORT1×HER2, bsSORT1×CTRL and bsCTRL×HER2 with DXd. We also prepared a DS8201a biosimilar (trastuzumab-DXd) as control. BsSORT1×HER2-DXd, bsSORT1×CTRL-DXd and bsCTRL×HER2-DXd had drug/antibody ratios (DARs) of 6.1, 6.5 and 6.1, respectively (Figure S1). The DAR of trastuzumab-DXd was 7.4 (Figure S2). After incubating with ADC, cell viability was assessed using a CCK8 assay. As shown in Figure 5A, bsSORT1×HER2-DXd induced significantly higher cytotoxicity over bsCTRLxHER2-DXd and trastuzumab-DXd and higher cytotoxicity over bsSORT1xCTRL-DXd, especially at lower concentrations. When using MDA-MB-231 (Figure 5B), the most commonly used cell line for breast cancer models, the cytotoxicity of bsSORT1xHER2-DXd was significantly better than all other ADCs. These results indicated that the co-targeting HER2 and SORT1 provided bsSORT1×HER2-DXd with superior cytotoxicity. On the contrary (Figure 5C), SK-OV-3 was more sensitive to trastuzumab-DXd due to the high expression of HER2. However, bsSORT1×HER2-DXd still had the same cell-killing activity as bsCTRL×HER2-DXd, and showed higher cytotoxicity than bsSORT1×CTRL-DXd. Taken together, bsSORT1×HER2-ADC has cytotoxicity against a wider range of applicable tumor types than single-target ADCs. Especially on HER2-low/SORT1-high tumors, bsSORT1×HER2-ADC will exhibit better activity than HER2-targeted ADCs.

### 2.6. BsSORT1×HER2-DXd Inhibited Tumor Growth In Vivo

**Figure 6:**
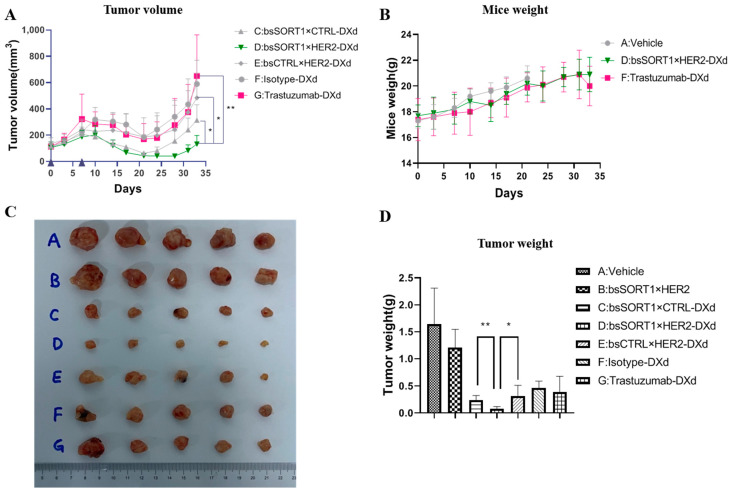
**Antitumor efficacy of bsADCs in MDA-MB-231 xenograft model.** The tumor-bearing mice were injected intraperitoneally with vehicle (PBS), bsSORT1×HER2, bsSORT1×CTRL-DXd, bsSORT1×HER2-DXd, bsCTRL×HER2-DXd, trastuzumab-DXd and isotype-DXd on day 0 and day 7. Tumor volume (**A**) and mice weight (**B**) were measured twice a week. The data are presented as mean ± SD from five mice. After termination, all tumors were taken for photographing (**C**) and weighing (**D**). *, *p* < 0.05; **, *p* < 0.001.

The in vivo activity of bsSORT1×HER2 ADC in HER2-low-expression tumors was investigated using an MDA-MB-231 xenograft model. When tumors reached an average size of approximately 150 mm^3^, mice were treated with 10 mg/kg ADCs on day 0 and day 7. As shown in Figure 6A, bsSORT1×HER2-DXd induced superior tumor suppression compared to trastuzumab-DXd and bsCTRL×HER2-DXd. The tumor growth rate of bsSORT1×HER2-DXd was similar to that of bsSORT1×CTRL-DXd in the first few days. But later the tumor size in the bsSORT1×HER2-DXd group was significantly smaller than that in the bsSORT1×CTRL-DXd group as well as at the end point. This result suggested that the advantages of bsSORT1×CTRL-DXd would become more prominent in the later stages. From Figure 6C,D, we found that all DXd-conjugated antibodies induced an inhibition of tumor growth, while bsSORT1×HER2 had minimum effect. The tumor size in the bsSORT1×HER2-DXd group was much smaller than in the other groups, and the weight of the tumors from the bsSORT1×HER2-DXd group was significantly lighter than that from the trastuzumab-DXd group. In addition, no significant change in mouse body weight was observed between the trastuzumab-DXd and bsSORT1×HER2-DXd groups (Figure 6B). This confirms that the involvement of a SORT1-targeted arm facilitates the endocytosis process of bsSORT1×HER2-DXd, leading to more tumor cell death in vivo.

## 3. Discussion

Currently, there are many ways to increase the inhibitory activity of HER2-targeted ADCs in HER2-low-expression tumors, for example increasing bystander killing activity, choosing a degradable linker or enhancing internalization [9,17,18]. In this study, we tried to enhance the activity of HER2-targeted ADCs by modifying the antibody part of the ADC to a bispecific antibody that can induce faster internalization of ADC. For this purpose, we needed to select a suitable target with faster internalization function and that co-expresses with HER2 on the tumor cell surface. Roselli S et al. [28] proved that the expression of SORT1 in breast cancer was significantly increased compared to normal tissue using immunohistochemistry experiments. Western blotting (WB) and quantitative RT-PCR data showed that the SORT1 levels in different breast cancer cell lines, including MCF-7, MDA-MB-231 and MDA-MB-468, were significantly higher than those in normal cells. We detected the surface SORT1 of different cancer cell lines using flow cytometry, and found that SORT1 expressed on the surface of the breast cancer cells MCF-7, MDA-MB-231, MDA-MB-468 and T47D, but did not express on the surface of the noncancer cell MCF-10A. We also observed that SORT1 co-expressed with HER2 in MCF-7, MDA-MB-231 and T47D. In addition, surface SORT1 triggers the internalization of its ligands from extracellular, suggesting the SORT1 antibody may also have faster internalization. Demeule M et al. [30] developed a SORT1-targeted peptide drug conjugate (PDC) for the treatment of SORT1-positive triple-negative breast cancer. We showed that more SORT1 antibodies were internalized by T47D than HER2 antibodies, even though T47D has more surface HER2 than SORT1. Taken together, SORT1 is an ideal target of HER2-targeted bsADCs due to its rapid internalization, co-expression with HER2 and ubiquitous expression on tumor cells for the treatment of HER2-low-expression tumors. 

There are many different bispecific antibody formats available to achieve desired functions and characteristics [33,34]. The bsSORT1×HER2 we constructed is based on a monovalent bispecific IgG format, which is monovalent for each antigen, while conventional IgG antibodies are bivalent. We used knobs-into-holes technology enabling FC heterodimerization to address the heavy-chain mispairing. The HER2-targeted arm is in the form of scFv to address heavy- and light-chain mispairing. Monovalent BsAbs are considered to have better targeting selectivity because they bind to a single target less actively than bivalent antibodies [16], thereby maximizing the killing of tumor cells that express two targets and reducing the risk to normal cells that only express a single target. In addition, we chose S82 as the SORT1-binding arm not only because of its rapid endocytosis activity, but also because of its relatively low affinity (Figure S3). Studies have shown that low-affinity binding arms can increase tumor specificity [20,35]. de Goeij BE et al. [20] selected a low-affinity anti-CD63 arm to enable bsHER2×CD63 selective binding and efficient internalization when a tumor expresses both HER2 and CD63. Likewise, Mazor Y et al. [36,37] evaluated a series of HER2/EGFR BsAbs with different EGFR affinities. They demonstrated that a BsAb with a reduced affinity to one target had better tumor selectivity in vivo, while higher affinity variants lost this selectivity.

Co-targeting two antigens can increase the tumor selectivity and improve the efficacy of bsADCs [16,38,39]. Vallera DA et al. [39] developed a CD19×CD22 bispecific immunotoxin (2219KDEL), and showed that the killing activity of 2219KDEL on CD19 and CD22 co-expressed cells was 150 times and 7.5 times higher than anti-CD19 scFv toxin and anti-CD22 scFv toxin, respectively. In addition, a series of EGFR×c-MET bsADCs developed by Sellmann C et al. [16] only showed superior killing activity over cetuximab-MMAE on tumor cells with a high expression of both EGFR and c-MET. Consistent with our expectation, the binding of bsSORT1×HER2 to a single target was obviously weaker than that of corresponding bivalent antibodies, but bsSORT1×HER2 showed superior binding capability on the SORT1 and HER2 co-expressed cell MDA-MB-231, which suggested that bsSORT1×HER2 had better binding selectivity for co-expressed cells. The higher binding activity of bsSORT1×HER2 indicated that bispecific binding enabled more antibodies to bind to cells, which may be useful to enhance the therapeutic benefit of ADCs. As a result, the cytotoxicity of bsSORT1×HER2-DXd on MDA-MB-231 or T47D was better than that of bsSORT1×CTRL-DXd and bsCTRL×HER2-DXd. Trastuzumab showed better cell-binding activity than bsSORT1×HER2 and bsSORT1×CTRL, but the cell-killing activity of trastuzumab-DXd was far lower than that of bsSORT1×HER2-DXd and bsSORT1×CTRL-DXd, which was consistent with their internalization properties. These results indicated that the internalization function may play a more important role in the killing activity of bsSORT1×CTRL-DXd on HER2-low-expression cells.

Improving the internalization activity of bsADCs by targeting rapid internalization antigens can also enhance their activity. Targeted CD63 was proved to be an ideal approach that facilitated internalization and lysosomal transport of HER2×CD63 BsAb (bsHER2×CD63) [20]. bsHER2×CD63 showed faster lysosomal transport than the HER2 monoclonal antibody, and its ADC had superior antitumor activity. However, CD63 is not only ubiquitously expressed on normal tissue, but also found to be frequently downregulated in tumors [40]. In addition, bsHER2×CD63-ADC only works on HER2-high-expression cell lines such as HCC1594 or SK-OV-3, but had minimum effect on the HER2-low-expression cell Colo205. In contrast, SORT1 expression in tumors is remarkably increased compared to normal tissue [28], which suggests that SORT1 may be a better tumor-specific target for promoting endocytosis. In this study, bsSORT1×HER2 showed rapid internalization because of SORT1 targeting. Unlike bsHER2×CD63, bsSORT1×HER2 resulted in more and faster degradation of HER2 in the HER2-low-expression cells T47D and MDA-MB-231, but did not show a similar effect on the HER2-high-expression cell SK-OV-3, which may be due to the lower expression of SORT1 in SK-OV-3. Compared to bsHER2×CD63-ADC, bsSORT1×HER2-DXd showed superior inhibition activity on HER2-low-expression cells, which may be due to the anti-SORT1 arm we chose inducing a stronger internalization effect than the anti-CD63 arm. Of course, the effect of bsSORT1×HER2 and bsSORT1×HER2-DXd on the HER2-high/SORT1-high-expression cell still needs further study. Similarly, Andreev J et al. [18] proved the concept by coupling HER2 with PRLR, a surface protein with high turnover. They developed a HER2×PRLR BsAb that showed faster endocytosis and rapid lysosomal degradation of HER2 in the HER2-low-expression cell T47D, and showed that the HER2×PRLR bsADC induced better growth inhibition of T47D, but they did not provide the tumor inhibition data for HER2×PRLR bsADC in vivo. However, we demonstrated in this study that bsSORT1×HER2-DXd not only possessed superior activity in vitro, but also showed better tumor inhibition in the MDA-MB-231 xenograft model. Of course, there are many other candidates with faster internalization that may be paired with HER2 for the development of bsADCs, for example tissue factor (TF), cation-independent mannose-6-phosphate receptor (CI-M6PR) and asialoglycoprotein receptor (ASGPR) [41,42].

Compared to T-DM1, DS-8201a was shown to have a wider therapeutic window and better safety profile. Furthermore, DS8201a has a significant inhibitory effect on HER2 low-expression tumors, on which T-DM1 has minimum effect [10], and now is approved for the treatment of HER2-low tumors. The cytotoxic payload and linker we used in this study are similar to DS-8201a. BsSORT1×HER2-DXd showed superior inhibitory activity over the DS-8201a biosimilar on HER2 low-expression tumors in vitro and in vivo. This proves that improving internalization using a bispecific antibody is an effective strategy to enhance the efficacy of HER2-targeted ADCs. A better activity of bsSORT1×HER2-DXd means that the same efficacy can be achieved at lower doses, and therefore may reduce the side effects associated with DXd, which are common in clinical usage of DS-8201a.

In summary, we have developed a SORT1×HER2 BsAb that possessed faster internalization and induced higher degradation of HER2 in HER2 low-expression tumors. On the one hand, SORT1 targeting plays a role in rapid endocytosis. On the other hand, HER2 targeting improves antibody binding activity and binding specificity. The features of the bsSORT1×HER2 enable its DXd conjugate (bsSORT1×HER2-DXd) to effectively kill tumor cells in vitro and in vivo. These results support that bsSORT1×HER2-DXd can be a promising HER2-targeting ADC.

## 4. Materials and Methods

### 4.1. Cell Lines

T47D (breast cancer), MDA-MB-231 (breast cancer), MDA-MB-468 (breast cancer), MCF-7 (breast cancer), MCF-10A (noncancer breast epithelial cell), A549 (lung carcinoma), SK-OV-3 (ovarian cancer), LNCAP (prostate carcinoma) and NCI-N87 (gastric carcinoma) cells were obtained from ATCC. T47D, LNCAP and NCI-N87 were cultured in RPMI-1640 (Hyclone, Logan, UT, USA) containing 10% FBS (Gibco, Waltham, MA, USA). MDA-MB-231, MDA-MB-468, A549 and SK-OV-3 were cultured in DMEM (Hyclone, USA) containing 10% FBS, and MCF-7 was cultured in MEM (Hyclone, USA) containing 10% FBS. MCF-10A was cultured in MCF-10A complete medium (Procell, Kowloon, China).

### 4.2. Antibodies and Conjugates

The sequences of SORT1 antibodies S02, S60 and S82 were acquired from patent US10428150B2. The heavy-chain and light-chain sequences of the HER2 antibodies trastuzumab and pertuzumab were acquired from international ImMunoGeneTics information system (IMGT). A nonbinding isotype antibody (CTRL) was used as the negative control in the study. Monovalent BsAb (bsSORT1×HER2) was constructed using the “knobs-into-holes” (KIH) approach [43]. The anti-SORT1 arm derived from S82 is in the form of a Fab format connecting FC domain in knobs, and the anti-HER2 arm from trastuzumab is in the form of an scFv format connecting FC domain in holes (Figure 3A). Using the same method, the corresponding control antibodies bsSORT1×CTRL and bsCTRL×HER2 were constructed, in which the anti-HER2 arm and anti-SORT1 arm were replaced by isotype controls, respectively. All antibody constructs were cloned into the mammalian expression vector pCDNA3.1 (Thermo Fisher, Waltham, MA, USA) for transient transfection and antibody expression in Expi293F (Thermo Fisher, USA). 

DXd-conjugated ADCs were generated via covalent conjugation of DXd (MCE, Monmouth Junction, NJ, USA) on antibody cystine groups as previously described [10]. After conjugation, the drug/antibody ratio (DAR) value of ADC was measured using reversed-phase liquid chromatography mass spectrometry (RPLC-MS) [44]. Fluorescein (FITC)-labeled pertuzumab was prepared by conjugating pertuzumab with FITC using NHS-Fluorescein (Thermo Fisher, USA).

### 4.3. Binding ELISA

For HER2 binding assay, 1 µg/mL HER2 extracellular domain (ECD) protein (Sino Biological Inc., Beijing, China) was directly coated on 96-well immunoplates and incubated overnight at 4 °C. The plates were blocked with 1% BSA for 1 h at 37 °C. For SORT1 protein coating, plates were firstly incubated with streptavidin (Sigma, Taufkirchen, Germany) overnight at 4 °C, and then were blocked with 1% BSA followed by incubation with 1 µg/mL biotin-labeled SORT1 ECD protein. After washing, serial-diluted antibody was added to the coated wells for 1 h at 37 °C. Then, the plates were washed and incubated with horseradish peroxidase (HRP)-conjugated anti-human IgG (Sigma, Germany) for 0.5 h at 37 °C. After washing, tetramethylbenzidine (TMB) solution (Sigma, Germany) was added and the reaction was stopped after 5 minutes using 1 M H_2_SO_4_. The A450 value of each well was measured with a microplate reader (Biotek, Seattle, WA, USA).

### 4.4. Flow Cytometry

For evaluation of the antigen level on cell surface, tumor cells were firstly blocked with 1% bovine serum albumin (BSA) on ice for 1 hour, followed by incubation with SORT1 antibody (S82), HER2 antibody (trastuzumab) or isotype control for 1 h. After washing, tumor cells were incubated with Alexa Fluor 647-conjugated goat anti-human IgG (Thermo Fisher, USA) for 0.5 h. Then, the mean fluorescence intensity (MFI) of cells was detected using flow cytometry (Beckman, USA) and depicted using FlowJo software 10 (BD Biosciences). The surface levels of SORT1 and HER2 were expressed as SORT1/ISO ratio and HER2/ISO ratio, respectively. SORT1/ISO ratio or HER2/ISO ratio were calculated with the following equation:

$$SORT1/ISO\;ratio\;or\;HER2/ISO\;ratio=\frac{MFI\;of\;anti―SORT1\;antibody\;or\;anti―HER2\;antibody}{MFI\;of\;isotype\;control}$$

For evaluation of cell-binding activity, MDA-MB-231 cells were incubated with serial diluted antibodies for 1 h, followed by incubation with Alexa Fluor 647-conjugated goat anti-Human IgG (Thermo Fisher, USA) for 0.5 h. The MFI was detected via flow cytometry (Beckman, Brea, CA, USA).

For the HER2 downmodulation assay, tumor cells were seeded in 24-well plates (Nunc, Roskilde, Denmark) and incubated overnight at 37 °C. Antibodies were added (20 nM) and cells were cultured for 24 h at 37 °C. Cell suspensions were obtained via trypsinization followed by blocking with 1% BSA on ice for 1 h. Then, tumor cells were incubated with FITC-labeled pertuzumab on ice for 0.5 h. After washing, MFIs of different groups were measured via flow cytometry. The group incubated with FITC-labeled pertuzumab but without antibody was used as the control. The relative HER2 levels were calculated with the following equation:

$$Relative\;HER2\;level=\frac{MFI\;of\;antibody-MFI\;of\;without\;FITC―labeled\;pertuzumab}{MFI\;of\;control-MFI\;of\;without\;FITC―labeled\;pertuzumab}\times 100\%$$

### 4.5. Internalization Measurement

The internalization of antibodies was detected following the instructions of pHrodo^TM^ iFL IgG labeling reagents (Thermo Fisher, USA). Briefly, tumor cells were seeded in 96-well plates (Nunc, Denmark) and incubated overnight at 37 °C. Antibodies were premixed with pHrodo^TM^ iFL green-labeled Fab fragments for 5 min, and then mixtures were added into plates and incubated for 1, 4, 10 or 24 h. After trypsinization, the MFI of tumor cells was measured using flow cytometry (Beckman, USA).

### 4.6. Western Blotting

After treatment with antibodies, tumor cells were lyzed with radioimmunoprecipitation assay (RIPA) buffer, and the cell lysates were resolved on 10% SDS-PAGE gel. The proteins on gel were transferred to PVDF membranes and then blocked with 5% nonfat dry milk for 1 hour. After washing, membranes were incubated overnight with HER2 antibody (ABclonal, Wuhan, China) and tubulin antibody (ABclonal, China). On the next day, membranes were washed and incubated with HRP-conjugated secondary antibody (ABclonal, China) for 1 h. After washing, HRP substrate (Bio-Rad, Hercules, CA, USA) was added onto membranes and signals were detected using chemiluminescence.

### 4.7. Cytotoxicity Assay

Tumor cells were seeded in 96-well tissue culture plates and then incubated with serially diluted ADC for 72 h at 37 °C. Cell viability was assessed using Cell Counting Kit-8 (CCK8) (Abbkine, Atlanta, GA, USA) according to the instructions. 

### 4.8. Tumor Xenograft Model

M-NSG mice (females, 6 weeks old) were procured from Shanghai Model Organisms Center, Inc. (Shanghai; China) and housed in pathogen-free conditions with free access to food and water. All animal experiments were approved by the Animal Ethical Committee at China State Institute of Pharmaceutical Industry and conducted according to the Code of Practice for the Housing and Care of Animals Used in Scientific Procedures. MDA-MB-231 (1 × 10^7^) in PBS containing 50% Matrigel was injected in the right-side flank of mice. When tumors reached an average size of approximately 150 mm^3^, animals were randomized into 7 groups. Each group consisted of 5 mice. ADCs were injected intravenously at a dose of 10 mg/kg once a week for 2 doses. Tumor sizes and mice weight were measured twice a week. Tumor volumes were calculated using the following equation: $tumor\;volume=\frac{\mathrm{length}\times {\mathrm{width}}^{2}}{2}$. After all experimental groups were terminated, tumors were taken for photographing and weighing. 

### 4.9. Statistical Analysis

All data analysis was performed using GraphPad Prism Software 8. Group data were expressed as mean ± SD. All p values were calculated using *t* tests, and group discrepancies were considered statistically significant when p values were less than 0.05.

## Figures and Tables

**Table 1:** The expression profile of HER2 and SORT1 on surface of tumor cell lines.

	HER2/ISO	SORT1/ISO	Expression Level
T47D	269.48	36.31	HER2-low/SORT1-high
MCF-7	135.94	15.38	HER2-low/SORT1-high
MDA-MB-231	70.21	33.15	HER2-low/SORT1-high
MDA-MB-468	1.10	17.11	HER2-neg/SORT1-high
MCF-10A	7.40	1.07	HER2-low/ SORT1-neg
LNCAP	137.86	7.79	HER2-low/SORT1-low
NCI-N87	3854.96	4.61	HER2-high/SORT1-low
SK-OV-3	1974.16	6.91	HER2-high/SORT1-low
A549	29.95	2.27	HER2-low/SORT1-low

## Data Availability

The data presented in this study are available on request from the corresponding author.

## Supplementary Materials

The following supporting information can be downloaded at https://www.mdpi.com/article/10.3390/ijms242216056/s1.

## Abbreviations

ADC	antibody–drug conjugate
BsAb	bispecific antibody
bsADC	bispecific antibody–drug conjugate
Fab	fragment antigen binding
FBS	fetal bovine serum
FC	fragment crystallizable region
FDA	U.S. Food and Drug Administration
IgG	immunoglobulin G
scFv	single-chain variable fragment
