# Supplementary material for: Generation of a Novel SORT1×HER2 Bispecific Antibody–Drug Conjugate Targeting HER2-Low-Expression Tumor
Source: Int J Mol Sci. 2023 Nov 7;24(22):16056. doi: 10.3390/ijms242216056 (PMC10671096; doi:10.3390/ijms242216056)
Supplement: Supplementary file 1 [file ijms-24-16056-s001.zip › ijms-2682222-supplementary.pdf]

Supplementary Materials

Supplementary Methods

DAR value detection

ADCs were deglycated using PNGase F for 10 minutes at 37°C, followed with DAR detection using RPLC-MS. RPLC was performed using MAbPac RP (Thermo Fisher) 4 µm 2.1×100mm on an Vanquish HPLC system (Thermo Fisher). MS was performed using Thermo Q Exactive HF-X system. The DAR of bsADC was calculated by the following equations:

$$\text{DAR(LC)} = \frac{\text{Intensity of LC(1 DXd)}}{\text{Total intensity of LC(0 DXd) and LC(1 DXd)}}$$

$$\text{DAR(Knob)} = \frac{\text{Intensity of Knob(1 DXd)}}{\text{Total intensity of Knob(0,1,2,3 DXd)}} + \frac{\text{Intensity of Knob(2 DXd)} \times 2}{\text{Total intensity of Knob(0,1,2,3 DXd)}} + \frac{\text{Intensity of Knob(3 DXd)} \times 3}{\text{Total intensity of Knob(0,1,2,3 DXd)}}$$

$$\text{DAR(Hole)} = \frac{\text{intensity of Hole(1 DXd)}}{\text{Total intensity of Hole(0,1,2,3 DXd)}} + \frac{\text{intensity of Hole(1 DXd)} \times 2}{\text{Total intensity of Hole(0,1,2,3 DXd)}} + \frac{\text{intensity of Hole(1 DXd)} \times 3}{\text{Total intensity of Hole(0,1,2,3 DXd)}}$$

$$\text{TOTAL DAR} = \text{DAR(LC)} + \text{DAR(Knob)} + \text{DAR(Hole)}$$

The DAR of trastuzumab-DXd was calculated by the following equations:

$$\text{DAR(LC)} = \frac{\text{Intensity of LC(1 DXd)}}{\text{Total intensity of LC(0 DXd) and LC(1 DXd)}}$$

$$\text{DAR(HC)} = \frac{\text{Intensity of HC(1 DXd)}}{\text{Total intensity of HC(0,1,2,3 DXd)}} + \frac{\text{Intensity of HC(2 DXd)} \times 2}{\text{Total intensity of HC(0,1,2,3 DXd)}} + \frac{\text{Intensity of HC(3 DXd)} \times 3}{\text{Total intensity of HC(0,1,2,3 DXd)}}$$

$$\text{TOTAL DAR} = 2 \times (\text{DAR(LC)} + \text{DAR(HC)})$$

Affinity measurement

The affinities of SORT1 antibodies were measured using an OCTET instrument (Sartorius). Antibodies (5µg/ml) were loaded on anti-human FC (AHC) sensors (Sartorius) for 240s. The sensors were then incubated with diluted SORT1 ECD protein for association for 180s and followed by placing the sensors into PBS for dissociations for 600s. The equilibrium dissociation constant (KD) was analyzed using Data Analysis 12.0 software (Sartorius).

Supplementary Figures

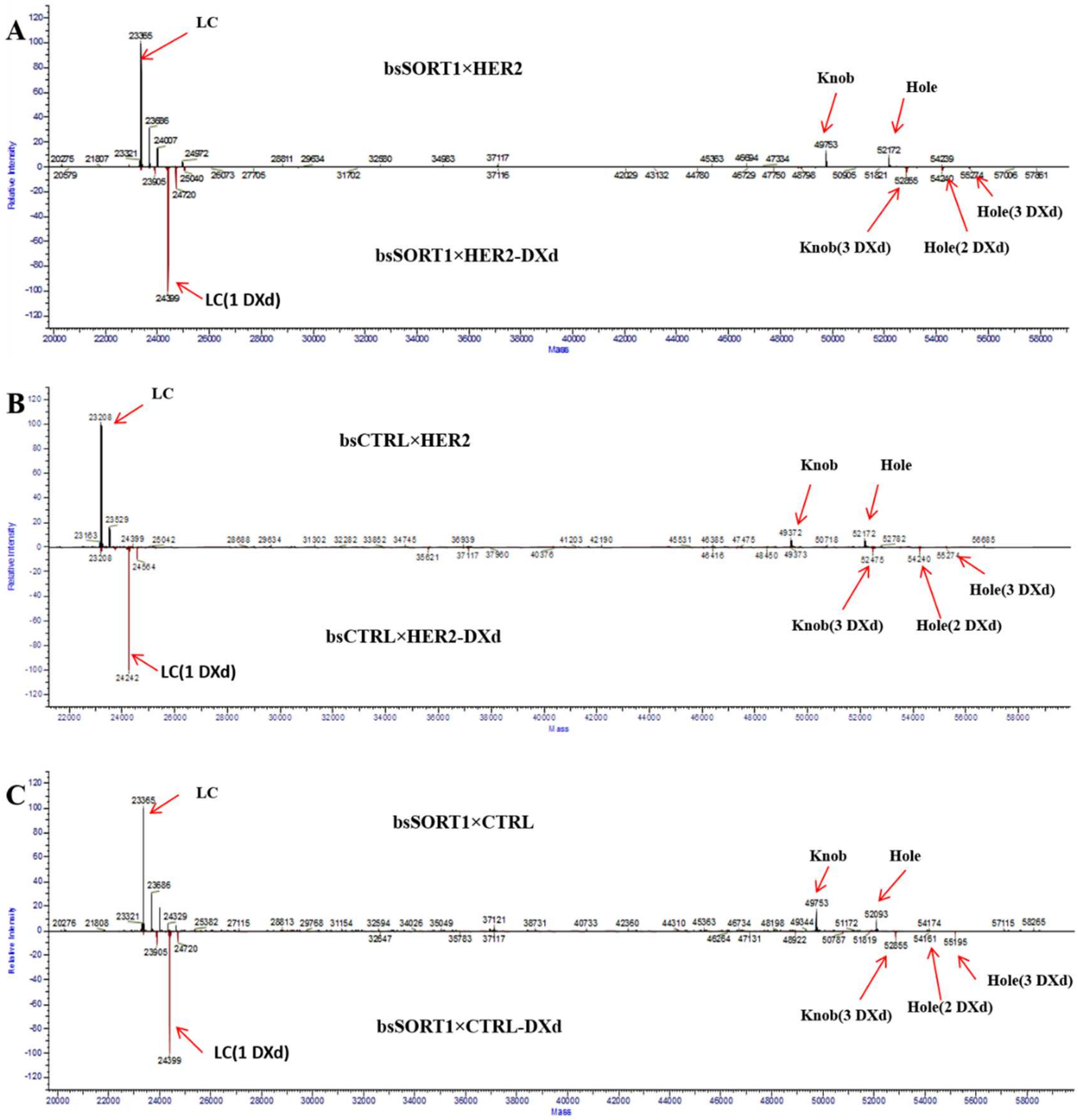

Figure S1. DAR measurement of bsSORT1×HER2-DXd (A), bsCTRL×HER2-DXd (B) and bsSORT1×CTRL-DXd (C)

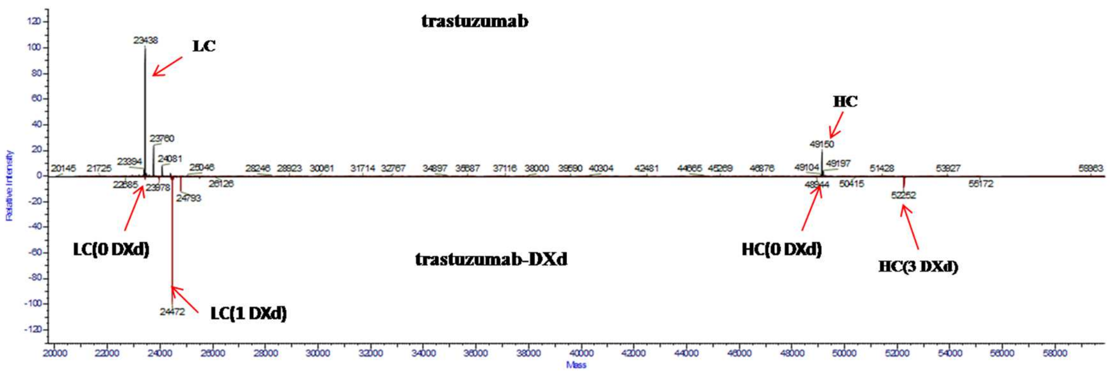

Figure S2. DAR measurement of trastuzumab-DXd.

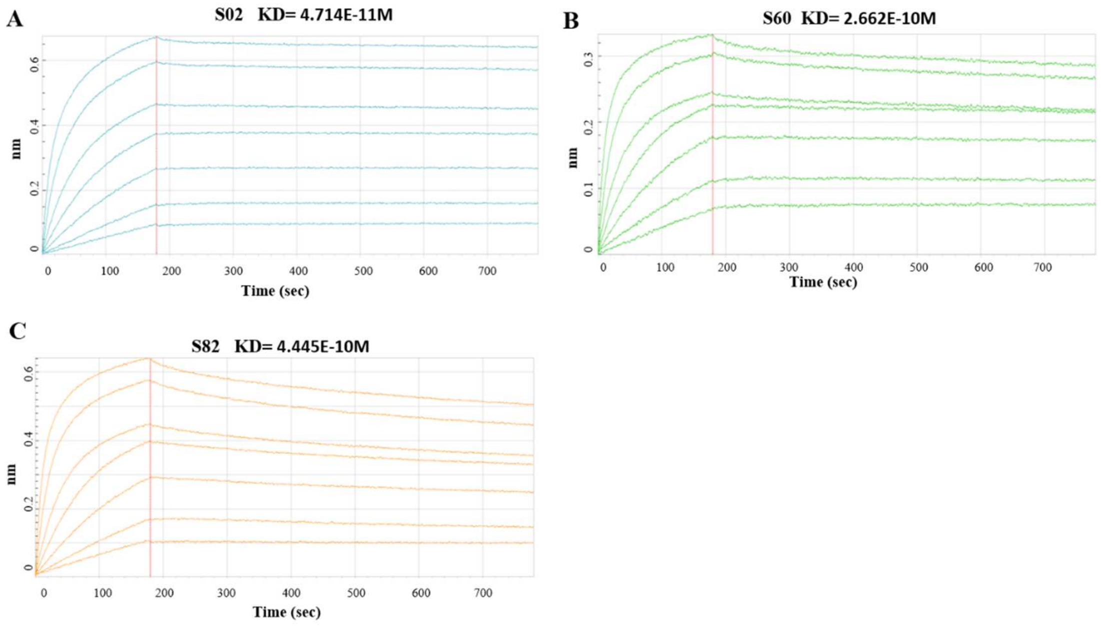

Figure S3. Affinity measurement of S02 (A), S60 (B) and S82 (C).
